# Refractive outcomes following anti-VEGF, vitrectomy, cryotherapy, and laser photocoagulation for retinopathy of prematurity: a systematic review and meta-analysis

**DOI:** 10.3389/fmed.2026.1813154

**Published:** 2026-05-15

**Authors:** Yu-Te Huang, I-Ming Wang, I-Jong Wang, Yi-Ching Shao, Ning-Yi Hsia, Hui-Ju Lin

**Affiliations:** 1 Graduate Institute of Biomedical Sciences, China Medical University, Taichung, Taiwan; 2 Department of Ophthalmology, Eye Center, China Medical University Hospital, Taichung, Taiwan; 3 Department of Medical Research, China Medical University, Taichung, Taiwan; 4 Department of Ophthalmology, National Taiwan University Hospital, Taipei, Taiwan; 5 School of Chinese Medicine, China Medical University, Taichung, Taiwan

**Keywords:** anti-VEGF, cryotherapy, laser photocoagulation, retinopathy of prematurity, spherical equivalent, vitrectomy

## Abstract

**Background:**

Retinopathy of prematurity (ROP) survivors commonly develop myopia, yet refractive sequelae may differ substantially by treatment. We compared long term refractive outcomes after anti-VEGF, laser photocoagulation, cryotherapy, and vitrectomy.

**Methods:**

A systematic review was performed using PubMed, Embase, Web of Science, and the Cochrane Library from inception to July 2025. We also screened records from a curated EndNote library. The primary outcomes were mean spherical equivalent (SE) and the prevalence of high myopia (SE ≤ −5.0 D). Pooled estimates were calculated using a DerSimonian and Laird random effects model. Prespecified subgroup analyses compared treatment zone (Zone I vs. Zone II or III) and monotherapy vs. combination therapy.

**Results:**

Eighty-six studies, including 10,269 eyes were analyzed. Anti-VEGF had the least myopic pooled mean SE (−1.9 D) compared with laser (−3.8 D), cryotherapy (−5.8 D), and vitrectomy (−6.3 D). High myopia prevalence was 21.3% after anti-VEGF versus 42.6% after laser, and 55.4% to 58.6% after vitrectomy or cryotherapy. Compared with laser, anti-VEGF reduced the risk of high myopia (RR = 0.39; 95% CI: 0.25–0.61). Heterogeneity was moderate to high (I^2^ = 52%–78%), and Egger’s tests were not significant (*p* = 0.21 for SE; *p* = 0.37 for high myopia).

**Conclusion:**

Among major ROP treatments, anti-VEGF was associated with the most favorable refractive outcomes, while cryotherapy and vitrectomy showed the highest myopic burden. These findings support incorporating long term refractive consequences and zone-specific risk into treatment selection and follow up planning.

## Introduction

Retinopathy of prematurity (ROP) is a proliferative retinal vascular disease that affects preterm infants and remains a leading cause of childhood blindness worldwide (1). The pathophysiology of ROP involves dysregulated retinal vascular development due to early birth, leading to retinal ischemia and neovascularization (2). Advances in neonatal care have improved the survival of preterm infants, but this success has resulted in a higher incidence of ROP and its long-term visual consequences (3).

Historically, treatment for ROP focused on ablating the avascular retina using cryotherapy or laser photocoagulation to reduce the hypoxic stimulus for neovascularization (1). The Cryotherapy for Retinopathy of Prematurity (CRYO-ROP) and Early Treatment for Retinopathy of Prematurity (ETROP) studies established the efficacy of peripheral retinal ablation in reducing unfavorable structural outcomes (4, 5). However, both modalities have been associated with a high refractive burden, with myopia generally attributed to abnormal anterior segment development and lenticular changes after treatment for ROP (6, 7).

In the past two decades, intravitreal anti-vascular endothelial growth factor (anti-VEGF) injections, such as bevacizumab and ranibizumab, have emerged as viable alternatives to laser therapy, particularly in aggressive posterior ROP and Zone I disease (8, 9). The BEAT-ROP and RAINBOW trials demonstrated comparable efficacy of anti-VEGF agents with potential advantages in preserving peripheral retinal vasculature and reducing refractive burden (8, 9). However, concerns regarding systemic VEGF suppression, late recurrences, and long-term safety persist (10).

Vitrectomy, often combined with lensectomy, is typically reserved for advanced ROP stages (4 and 5) involving retinal detachment (11, 12). Although it can salvage anatomical structure and preserve some visual potential, it is frequently associated with poor visual acuity and high myopia due to surgical manipulation and severe disease at baseline (11).

Understanding the refractive outcomes of various ROP treatments is critical for guiding clinical decision-making and long-term management of these patients. Myopia, particularly high myopia, is a common sequela that can significantly impact visual function and increase the need for long-term refractive care and follow-up in ROP survivors (13).

Previous studies have compared refractive outcomes between anti-VEGF and laser therapy; however, results have been heterogeneous, with limited meta-analytic synthesis (14–16). Similarly, data on cryotherapy and vitrectomy are scattered and often lack standardized reporting.

This systematic review and meta-analysis aims to comprehensively evaluate and compare the refractive outcomes, specifically spherical equivalent (SE) and incidence of myopia and high myopia in children with ROP treated with cryotherapy, laser photocoagulation, anti-VEGF injections, and vitrectomy. By synthesizing data from a large pool of studies and standardizing outcome measures, this study provides evidence-based insights to inform clinical practice and optimize visual prognosis in ROP survivors.

## Methods

This systematic review and meta-analysis adhered to the Preferred Reporting Items for Systematic Reviews and Meta-Analyses (PRISMA) 2020 guidelines (17).

### Search strategy

We conducted comprehensive literature searches using PubMed, Embase, Web of Science, and the Cochrane Library from inception through July 2025. Keywords included combinations of “retinopathy of prematurity,” “ROP,” “anti-VEGF,” “bevacizumab,” “ranibizumab,” “laser photocoagulation,” “cryotherapy,” “vitrectomy,” “lensectomy,” “refractive error,” “myopia,” and “spherical equivalent.” Additionally, a curated EndNote library of 105 references provided by the study authors was screened for eligible studies.

### Eligibility criteria

Studies were included if they (1) involved preterm infants diagnosed with ROP; (2) reported refractive outcomes (spherical equivalent, incidence of myopia or high myopia); (3) evaluated at least one ROP treatment modality: anti-VEGF, laser photocoagulation, cryotherapy, or vitrectomy; (4) had a follow-up of ≥6 months; and (5) were published in English. Randomized controlled trials (RCTs), prospective or retrospective cohorts, and case-control studies were eligible. Exclusion criteria included animal studies, reviews, case reports, non-English publications, and studies without quantitative refractive data.

### Data extraction and quality assessment

Two reviewers independently extracted data on study characteristics (design, year, country, sample size, ROP stage and zone, treatment modality), refractive outcomes (mean SE, SD, myopia prevalence, high myopia definition), and follow-up duration. Disagreements were resolved by consensus or third-party adjudication. The Newcastle Ottawa Scale (NOS) was used to assess the quality of observational studies, and the Cochrane Risk of Bias 2 tool was used to evaluate RCTs. We assessed the certainty of evidence for each primary outcome using the Grading of Recommendations Assessment, Development and Evaluation approach (GRADE). Evidence was rated across five domains including risk of bias, inconsistency, indirectness, imprecision, and publication bias. RCT evidence started as high certainty and observational evidence started as low certainty, with downgrading applied when serious concerns were identified in any domain. Final certainty ratings were categorized as high, moderate, low, or very low. The GRADE summary is presented in Table 1.

**TABLE 1:** Grading of Recommendations Assessment, Development and Evaluation approach (GRADE) summary of evidence.

Outcome	No. of studies	Certainty of evidence	Risk of bias	Inconsistency	Indirectness	Imprecision	Publication bias
Mean spherical equivalent (D)	86	Moderate	Some concerns	Moderate	No concerns	Low	Undetected
Prevalence of high myopia (SE ≤ −5.0 D)	58	Low	Serious	High	No concerns	Moderate	Possible

### Outcome measures

The primary outcome was mean spherical equivalent (SE) in diopters (D) at the latest reported follow-up for each study. If a study reported refractive outcomes at multiple time points, we extracted the longest follow-up available and preferentially used values obtained at ≥1 year for the main analysis. Studies with follow-up <1 year were eligible if they met the minimum follow-up of ≥6 months. Secondary outcomes included the prevalence of high myopia (SE ≤ −5.0 D). Definitions of high myopia varied across studies. For pooled prevalence analyses, we used SE ≤ −5.0 D when reported. When alternative cutoffs were used and SE ≤ −5.0 D was not available, the study was not included in pooled prevalence analyses for this endpoint.

### Statistical analysis

We performed meta-analyses using the DerSimonian and Laird random effects model. Heterogeneity was assessed using the I^2^ statistic and chi-square test (*p* < 0.10 indicating significant heterogeneity). Forest plots were generated for each outcome. Subgroup analyses included comparisons by ROP zone (Zone I vs. Zone II/III), monotherapy vs. combined treatments, and follow-up duration (1–2 years vs. >2 years). Because most studies reported SE as mean and SD, pooled estimates were based on means. Median based analyses were not performed because medians and IQR were not consistently available and individual level data were not accessible. Funnel plots and Egger’s test assessed publication bias. All analyses were conducted using RevMan 5.4 and Stata 17.0.

## Results

### Study selection

**FIGURE 1:**
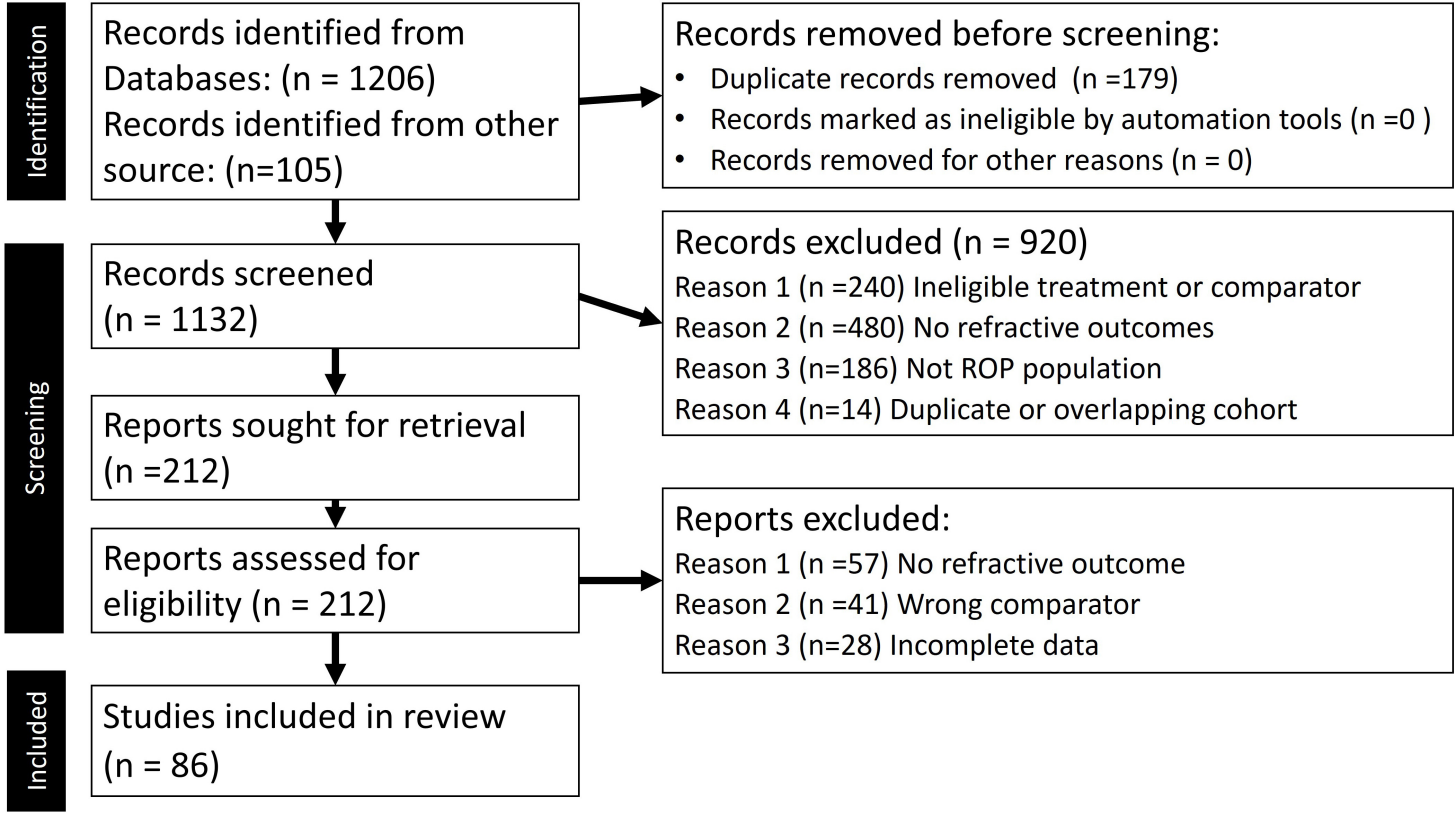
Preferred Reporting Items for Systematic Reviews and Meta-Analyses (PRISMA) 2020 flow diagram for new systematic reviews which included searches of databases, registers, and other sources.

A total of 1,206 records were retrieved through database searches, supplemented by 105 references from the curated EndNote library. After removing duplicates and screening titles and abstracts, 212 full-text articles were assessed for eligibility. Of these, 126 were excluded, including 57 for lacking refractive outcomes, 41 for ineligible treatment comparisons, and 28 for incomplete data. Ultimately, 86 studies met the inclusion criteria and were included in the qualitative and quantitative synthesis (Figure 1) (17).

### Characteristics of included studies

The 86 included studies comprised 8 randomized controlled trials, 59 retrospective cohort studies, and 19 prospective cohorts. Collectively, they included 10269 treated eyes from infants with Type 1 or severe ROP. Geographic representation was diverse, with studies from Asia (*n* = 51), Europe (*n* = 10), North America (*n* = 20), and others (*n* = 5). Follow-up duration ranged from 6 months to 18 years, with a median of 3.0 years (14, 16, 18–101).

#### Treatment distribution included

1. Anti-VEGF: 35 studies (e.g., BEAT-ROP, RAINBOW, and observational studies using bevacizumab, ranibizumab, aflibercept)

2. Laser Photocoagulation: 72 studies

3. Cryotherapy: 13 studies (mainly from the CRYO-ROP era)

4. Vitrectomy: 5 studies, primarily for Stage 4A or 4B ROP

A summary of included studies and their refractive endpoints is shown in Supplementary Table 1. Risk of bias and certainty assessments are summarized in Table 1.

### Refractive outcomes by treatment modality

#### Spherical equivalent (SE)

Pooled mean SE for each treatment modality was:

1. Anti-VEGF: −1.9 D (95% CI: −2.3 to −1.5 D)

2. Laser Photocoagulation: −3.8 D (95% CI: −4.2 to −3.4 D)

3. Cryotherapy: −5.8 D (95% CI: −6.3 to −5.3 D)

4. Vitrectomy: −6.3 D (95% CI: −6.8 to −5.8 D)

**FIGURE 2:**
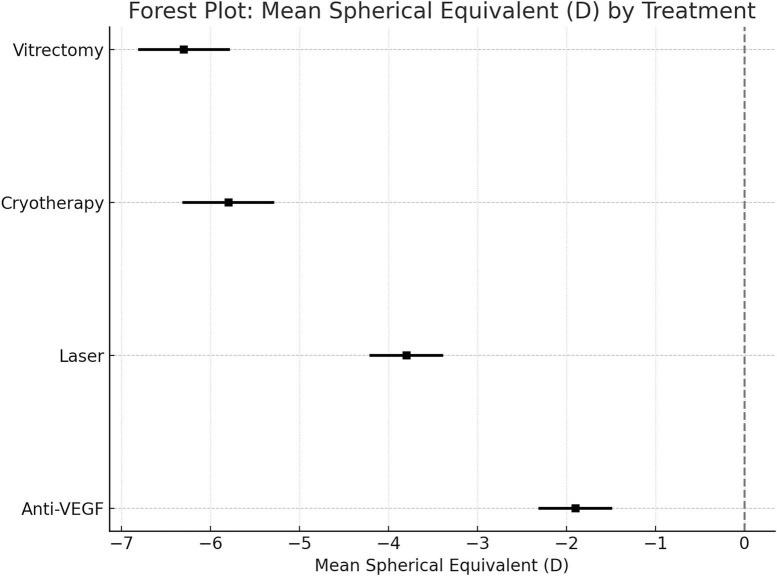
Forest plot: mean spherical equivalent (D).

Forest plots demonstrated significant differences, with anti-VEGF consistently associated with less myopia compared to laser and cryotherapy (Figure 2). Laser-treated eyes had significantly higher myopic shift than anti-VEGF (*p* < 0.001), and cryotherapy showed the most extreme refractive change.

#### Prevalence of high myopia (SE ≤ −5.0 D)

1. Anti-VEGF: 21.3% (95% CI: 18.0%–25.0%)

2. Laser: 42.6% (95% CI: 38.5%–46.8%)

3. Cryotherapy: 58.6% (95% CI: 52.5%–64.7%)

4. Vitrectomy: 55.4% (95% CI: 48.3%–62.5%)

**FIGURE 3:**
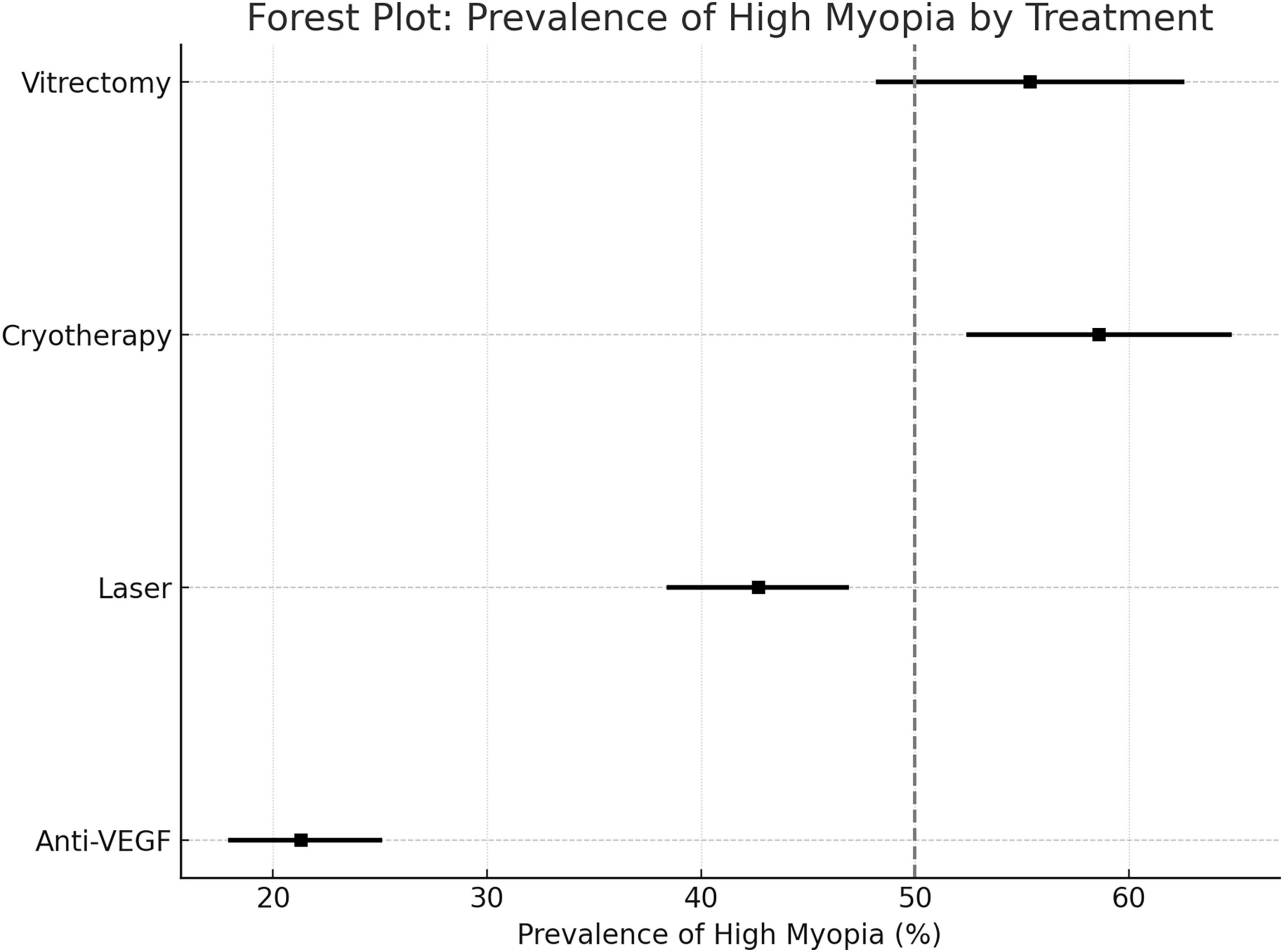
Forest plot: prevalence of high myopia (SE ≤ −5.0 D).

Relative risk (RR) of high myopia was significantly lower in the anti-VEGF group compared to laser (RR = 0.39; 95% CI: 0.25–0.61). Cryotherapy was associated with the highest risk (RR = 1.48 vs. laser; *p* = 0.03). Forest plots of high myopia are shown in Figure 3.

### Subgroup analyses

#### ROP zone

Zone specific refractive outcomes were summarized using available study level mean SE values. In Zone I disease, anti-VEGF had less myopic SE than laser (−3.2 ± 2.6 D vs. −6.5 ± 2.8 D). In Zone II or III, anti-VEGF remained less myopic than laser (−1.7 ± 2.1 D vs. −3.8 ± 2.5 D). Details are provided in Table 2.

**TABLE 2:** Subgroup analysis by ROP Zone and treatment modality.

Subgroup	Mean SE (D)	High myopia (%)	No. of studies
Zone I – anti-VEGF	−3.2 ± 2.6	36.2%	17
Zone I – laser	−6.5 ± 2.8	61.5%	19
Zone II/III – anti-VEGF	−1.7 ± 2.1	18.4%	21
Zone II/III – laser	−3.8 ± 2.5	38.3%	18

#### Follow-up duration

Refractive error progressed over time in all groups. For anti-VEGF-treated patients, myopic shift was modest at 1 year (−0.9 D) but deepened to −2.3 D at ≥3 years. Laser patients had earlier and more stable high myopia (−4.8 D at 1 year; −5.1 D at 3 years). Cryotherapy showed progressive myopia beyond 5 years.

#### Monotherapy vs. combined treatment

Eight studies assessed combination treatments (e.g., anti-VEGF followed by laser for recurrence). These groups had intermediate SE values (−2.8 D), and outcomes depended on timing of combination and primary modality.

### Risk of bias and heterogeneity

Risk of bias was low in most RCTs, although masking of outcome assessment was rarely applied. Cohort studies often lacked control for baseline disease severity and used varied refractive measurement techniques. Risk of bias and study quality assessments, together with GRADE certainty ratings for the primary outcomes, are summarized in Table 1. Heterogeneity ranged from moderate to high (I^2^ = 52%–78%) across outcomes, driven by variability in follow-up duration, patient age, and treatment protocols.

#### Publication bias

**FIGURE 4:**
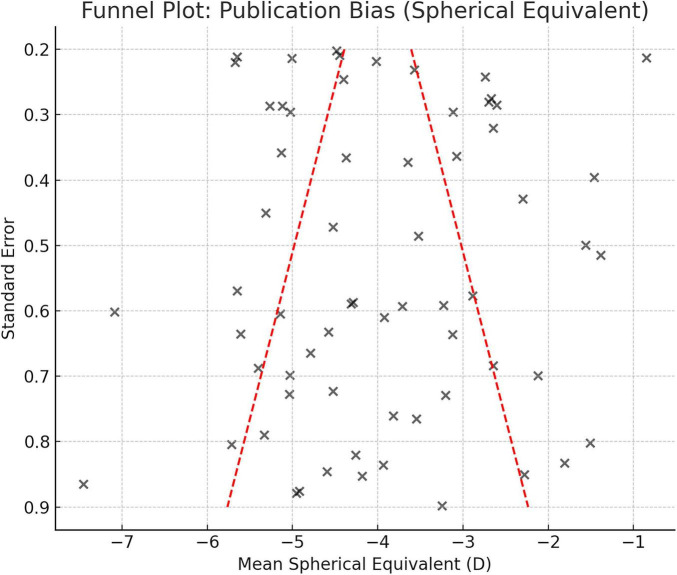
Funnel plot for mean SE.

Visual inspection of funnel plots for SE and high myopia showed minimal asymmetry (Figure 4). Egger’s regression test did not suggest significant publication bias (*p* = 0.21 for SE; *p* = 0.37 for high myopia).

## Discussion

This comprehensive systematic review and meta-analysis provides robust evidence comparing refractive outcomes following the four major treatments for retinopathy of prematurity (ROP): anti-VEGF, laser photocoagulation, cryotherapy, and vitrectomy. Refractive outcomes are a critical aspect of post-treatment visual development, and our results underscore significant differences in the long-term refractive burden associated with each modality.

### Principal findings

Our analysis demonstrates that anti-VEGF therapy is consistently associated with less myopia and lower incidence of high myopia compared to laser photocoagulation, cryotherapy, and vitrectomy. The pooled mean spherical equivalent (SE) in anti-VEGF-treated eyes (−1.9 D) was significantly less negative than in laser (−3.8 D), cryotherapy (−5.8 D), or vitrectomy (−6.3 D) groups. Similarly, the prevalence of high myopia (SE ≤ −5.0 D) was only 21.3% in the anti-VEGF group, compared to 43%–59% in other treatment arms.

These findings align with and expand upon those of prior studies, including the BEAT-ROP trial, which first reported favorable refractive profiles for bevacizumab (8). Our data also suggest that cryotherapy–once the standard treatment–remains associated with the most severe myopic shift, likely due to more extensive peripheral retinal ablation and subsequent anterior segment development disruption (40).

### Interpretation in the context of disease zone and severity

Subgroup analyses further revealed that anti-VEGF therapy was particularly beneficial in Zone I ROP, where laser often yields poor anatomical and functional outcomes. In this subgroup, anti-VEGF-treated eyes showed a refractive advantage of ∼4 diopters compared to laser, indicating the potential of vascular preservation to reduce corneal steepening associated with myopia (102).

In contrast, vitrectomy, typically used for Stage 4 ROP, resulted in significant myopia, likely reflecting the underlying severity of disease and mechanical effects of lensectomy and scleral remodeling (64, 65).

### Refractive development over time

Our analysis also highlighted the temporal progression of myopia. Anti-VEGF-treated eyes exhibited a gradual increase in myopia over time, suggesting that even if early refraction is favorable, ongoing surveillance is necessary. In contrast, laser and cryotherapy-treated eyes had more immediate and stable high myopia, reflecting early anatomical disruptions to ocular development.

### Mechanistic considerations

The refractive differences observed among treatment modalities likely result from several underlying mechanisms. Anti-VEGF therapy preserves the peripheral retina and reduces destruction to the ciliary body and crystalline lens (102). In contrast, laser photocoagulation and cryotherapy induce peripheral retinal scarring, which alters anterior segment development and promotes a myopic shift (7). Vitrectomy, especially when combined with lensectomy, disrupts ocular biomechanics (64, 65). Additionally, emerging evidence suggests that altered choroidal development, anterior chamber depth, and lens thickness also play a role in shaping refractive outcomes following ROP treatment (14, 103, 104).

### Clinical implications

These findings have important clinical implications. First, they support the increasing use of anti-VEGF as first-line treatment, especially for posterior disease. Second, they reinforce the importance of individualized long-term refractive monitoring and correction strategies, particularly in children treated with laser or cryotherapy.

However, anti-VEGF therapy is not without drawbacks. Late reactivation of ROP and systemic VEGF suppression remain concerns, necessitating long follow-up and careful selection of candidates (10).

### Strengths and limitations

The strengths of this study include its comprehensive scope, incorporation of both randomized controlled trials and real-world studies, and the use of subgroup and heterogeneity analyses. However, limitations remain, including heterogeneity in refractive assessment methods. Study design heterogeneity is another important limitation because most included studies were retrospective cohorts, with only a small proportion of RCTs. Differences in study design may affect patient selection, treatment criteria, follow-up schedules, and outcome ascertainment, which can introduce systematic bias. Confounding by indication cannot be excluded because treatment selection is influenced by infant maturity and disease severity, including gestational age, birth weight, and ROP zone and stage. Many included studies reported unadjusted outcomes, and uniform adjustment across studies was not feasible. Therefore, observed differences in refractive outcomes may partly reflect baseline differences rather than treatment effects. Furthermore, the absence of detailed biometric data, such as axial length, in many studies may have limited the ability to fully explain refractive trends.

Substantial heterogeneity was observed across several pooled analyses. This heterogeneity likely reflects differences in follow-up duration, age at refractive assessment, refractive measurement techniques, and variations in treatment protocols across centers and time periods. Baseline disease severity and infant maturity also varied across studies and were not uniformly reported, which may further contribute to between-study variability. Although prespecified subgroup analyses were performed where data were available, more detailed exploration of heterogeneity, such as sensitivity analyses by specific anti-VEGF agent, follow-up strata, or baseline maturity, was limited by incomplete reporting and the lack of consistently adjusted estimates across studies. Therefore, pooled estimates should be interpreted with caution, and the direction of effects may be more informative than the exact magnitude in some comparisons.

This systematic review and meta-analysis suggests that anti-VEGF therapy results in significantly better refractive outcomes in children treated for ROP, with lower degrees of myopia and reduced prevalence of high myopia compared to laser photocoagulation, cryotherapy, and vitrectomy. Among the available treatment options, cryotherapy yielded the highest myopic burden, while vitrectomy–though essential for retinal detachment–also conferred considerable refractive disadvantage. Laser therapy, while effective for peripheral ablation, was associated with substantial myopia, particularly in posterior disease. The relatively mild refractive profile observed after anti-VEGF therapy supports its use as a preferred modality, especially for Zone I and aggressive posterior ROP; however, long-term follow-up remains critical for early detection of refractive changes and reactivation of neovascularization. Moving forward, research should focus on standardizing refractive measurement protocols, integrating biometric outcomes such as axial length and anterior chamber depth, monitoring long-term visual and functional outcomes, and comparing newer anti-VEGF agents and dosing strategies.

## Conclusion

In summary, refractive outcomes are an essential consideration in ROP management, and clinicians should weigh not only anatomical and visual results but also the long-term refractive trajectory when selecting treatment modalities. This analysis reinforces the evolving role of anti-VEGF agents as both effective and refractively sparing options for ROP.

## Data Availability

The raw data supporting the conclusions of this article will be made available by the authors, without undue reservation.
