# Supplementary material for: Refractive outcomes following anti-VEGF, vitrectomy, cryotherapy, and laser photocoagulation for retinopathy of prematurity: a systematic review and meta-analysis
Source: Front Med (Lausanne). 2026 May 15;13:1811613. doi: 10.3389/fmed.2026.1813154 (PMC13219334; doi:10.3389/fmed.2026.1813154)
Supplement: Supplementary file 1 [file Table_1.docx]

**Supplementary 1: Characteristics of All Included Studies (n=90)**

| First Author (Year) | | Country | | Study Design | Treatment Modality | Sample Size (Eyes) | Mean Follow-up (yrs) | Mean SE (D) | High Myopia Prevalence (%) |
| --- | --- | --- | --- | --- | --- | --- | --- | --- | --- |
| Choi MY (2000)^18^ | | Korea | | Retrospective | Cryotherapy | 125 | 6 | -2.23 ± -6.97 | 25.6 |
| Kaur SD (2021)^19^ | | India | Prospective | Cryotherapy | 243 | 1 | -2.9 ± 4.3 | NA |  |
| Cerman E (2016) ^20^ | | Turkey | Retrospective | Cryotherapy | 46 | 1 | -1.76±2.69 | NA |  |
| Nguyen HT (2015)^21^ | | Vietnam | | Prospective | Laser | 100 | 5 | -2.75 | 32 |
| Kaur S (2017)^22^ | | India | | Prospective | Laser | 36 | 1 | -4.05 | 52.8 |
| Lenis TL (2020)^23^ | | USA | | Prospective | Laser | 50 | 5-15 | −0.88 | NA |
| Katoch D (2011)^24^ | | India | | Retrospective | Laser | 36 | 1 | -2.77 | 1.4 |
| Mori Y (2021)^25^ | | Japan | | RCT | Laser | 56 | 5.0 | −0.5 ± 3.0 | NA |
| [Alqurashi](https://pubmed.ncbi.nlm.nih.gov/?term=Alqurashi+L&cauthor_id=38435111) L (2023)^26^ | | Saudi Arabia | | Retrospective | Laser | 260 | 2.6 | +2.2± 4.58, +1.9±2.03, +2.7 | NA |
| Ruan LM (2016)^27^ | | China | | Prospective | Laser | 115 | 3.3 | -5.1, -4.1 | 2, 67 |
| Zeng X (2021)^28^ | | China | | Retrospective | Laser | 50 | 3-11 | -1.23, -0.07 | NA |
| Essex RW (2005)^29^ | | Australia | Retrospective | Laser | 107 | 2 | −0.6 | NA |  |
| Ells AL (2017)^30^ | | Canada | Retrospective | Laser | 21 | 1 | +1.00 | NA |  |
| Gunn DJ (2013)^31^ | | Australia | Retrospective | Laser | 128 | 18 | −2.25 ± 5.75 | 32 |  |
| [Jossy](https://pubmed.ncbi.nlm.nih.gov/?term=Jossy+A&cauthor_id=36760960) A (2022)^32^ | | India | Prospective | Laser | 51 | 2 | + 0.14 ± 2.50 | NA |  |
| Hwang ES (2022)^33^ | | USA | Retrospective | Laser | 153 | 3 | -0.8 | NA |  |
| Gunay M (2015)^34^ | | Turkey | Retrospective | Laser | 202 | 1 | 0.00 ± 2.48 | 14.3 |  |
| Kieselbach GF (2006)^35^ | | Austria | Retrospective | Transscleral or transpupillary laser | 37 | 5 | -1.0± 4 | NA |  |
| Lee GA (2004)^36^ | | Australia | Retrospective | Laser | 49 | 6.5 | -0.40 | 15.0 |  |
| Li ML (2013)^37^ | | Taiwan | Retrospective | Laser | 50 | 2.5 | −3.50 ± 4.27 | 34 |  |
| [Al-Otaibi](https://pubmed.ncbi.nlm.nih.gov/?term=Al-Otaibi+AG&cauthor_id=23961009) AG (2012)^38^ | | Saudi Arabia | Retrospective | Laser | 114 | 1-10 | −3.7 ± 6.5 | 28.9 |  |
| [Connolly](https://pubmed.ncbi.nlm.nih.gov/?term=Connolly+BP&cauthor_id=10485543) B P (1999)^39^ | | USA | Retrospective | Laser | 30 | 3.7 | −4.8 | NA |  |
| [Axer-Siegel](https://pubmed.ncbi.nlm.nih.gov/?term=Axer-Siegel+R&cauthor_id=18536600) R (2008)^40^ | | Israel | Retrospective | Laser | 194 | 5 | −1.5 ± 4.2 | 23.9 |  |
| Bayramoglu SE (2022)^41^ | | Turkey | Retrospective | Laser | 17 | 3 | 2.1 ± 2.5 | NA |  |
| Fallaha N (2002)^42^ | | USA | Retrospective | Laser | 91 | 3 | -4.52 ± 5.63 | NA |  |
| Gonzalez VH (2010)^43^ | | USA | Retrospective | Laser | 100 | 1 | -3.80 | NA |  |
| [Gursoy](https://pubmed.ncbi.nlm.nih.gov/?term=Gursoy+H&cauthor_id=24738951) H (2014)^44^ | | Turkey | Prospective | Laser | 23 | 1.5 | -1.46 ± 4.02 | NA |  |
| Halan P (2020)^45^ | | India | Prospective | Laser | 64 | 1 | -1.82 | 18.8 |  |
| Matsumura S (2022)^46^ | | Japan | Retrospective | Laser | 67 | 3 | −1.72 ± 3.53 | 12 |  |
| McLoone EM (2006)^47^ | | Ireland | Retrospective | Laser | 16 | 11 | -2.33 | NA |  |
| Anilkumar SE (2019)^48^ | | India | Retrospective | Laser | 80 | 6 | -5.29 ±  4.9 | NA |  |
| Yang CS (2010)^49^ | | Taiwan | Retrospective | Laser | 60 | 7 | -3.87 | NA |  |
| [Stoica](https://pubmed.ncbi.nlm.nih.gov/?term=Stoica+F&cauthor_id=28461831) F (2016)^50^ | | Romania | Retrospective | Laser | 96 | 0.5 | -4.12 | NA |  |
| Sayman Muslubas I (2017)^51^ | | Turkey | Prospective | Laser | 48 | 4-10 | 1.9 ± 2.5 | NA |  |
| Paysse EA (2007)^52^ | | USA | Retrospective | Pulsed versus near-continuous laser | 90 | 0.5 | −2.77  vs −3.78 | NA |  |
| Roohipourmoallai R (2022)^53^ | | USA | Prospective | transpupillary or transscleral laser | 209 | 0.5 | 0.31 ± 3.57 vs 0.44 ± 2.85 | 4.7 vs 4.8 |  |
| Isaac M (2023)^54^ | | Canada | Retrospective | Bevacizumab | 56 | 3 | -1.98 ± 4.91 | 8.9 |  |
| Yulia DE (2022)^55^ | | Indonesia | Prospective | Bevacizumab | 7 | 1 | -2.25, -7 | 25 |  |
| Etezad Razavi (2020)^56^ | | Iran | Retrospective | Bevacizumab | 38 | 1 | +1.34 ± 1.01 | NA |  |
| Fan YY (2019)^57^ | | Taiwan | Retrospective | Bevacizumab | 38 | 3 | 0.13 | NA |  |
| Larranaga-Fragoso P (2016)^58^ | | Spain | Retrospective | Bevacizumab | 28 | 1 | 1.99 | NA |  |
| Cheng H (2023)^59^ | | China | | Prospective | Ranibizumab | 204 | 3.5 | +1.5 | NA |
| [Martínez-Castellanos](https://pubmed.ncbi.nlm.nih.gov/?term=Mart%C3%ADnez-Castellanos+MA&cauthor_id=23099498)  MA (2013)^60^ | | Mexico | | Prospective | Ranibizumab | 13 | 4.5 | −0.5 to −6.75 | 7 |
| Meng Q (2020)^61^ | | China | | Retrospective | Ranibizumab | 186 | 2 | -0.17 | 0.6 |
| Chen YT (2020)^62^ | | Taiwan | Prospective | Aflibercept | 17 | 1 | -1.94±2.97 | NA |  |
| Salman A (2015)^63^ | | Egypt | RCT | Aflibercept | 26 | 1 | 0.75 | 3.8 |  |
| Macor S (2021)^64^ | | Italy | | Retrospective | Vitrectomy | 10 | 4 | –11.25 | 80 |
| Iwahashi C (2023)^65^ | | Japan | Retrospective | Vitrectomy | 42 | 4 | -10.1 ± 5.0 | 75.5 |  |
| Carvounis PE (2010)^66^ | | USA | Retrospective | Vitrectomy vs control | 9 | 3.9 | -6.78 vs -10.33 | NA |  |
| White JE (1997)^67^ | | USA | RCT | Cryotherapy vs laser | 19 | 3 | -7.62 vs -6.60 | NA |  |
| Shalev B (2001)^68^ | | USA | RCT | Cryotherapy vs laser | 19 | 7 | -8.25 vs -6.50 | NA |  |
| Paysse EA (1999)^69^ | | USA | Retrospective | Cryotherapy versus transpupillary laser | 128 | 1 | –3.28 vs–2.95 | NA |  |
| Wu WC (2013)^70^ | | Taiwan | Retrospective | Laser or cryotherapy vs control | 49 | 1 | -3.5 vs -1.1 | NA |  |
| [Connolly](https://pubmed.ncbi.nlm.nih.gov/?term=Connolly+BP&cauthor_id=10485543) B P (2002)^71^ | | USA | RCT | Cryotherapy vs laser | 118 | 10 | -7.65 vs -4.48 | NA |  |
| Al-Ghamdi A (2004)^72^ | | Canada | Retrospective | Cryotherapy vs laser | 71 | 3 | -9.21 vs. -1.80 | NA |  |
| Laws F (1997)^73^ | | England | Prospective | Cryotherapy vs laser | 34 | 1 | -5.25 vs -0.5 | NA |  |
| Marinov VG (2024)^74^ | | Bulgaria | | Retrospective | Laser or cryotherapy | 319 | 12 | NA | NA |
| Autrata R (2008)^75^ | | Czech | Retrospective | Cryotherapy+laser | 172 | 4 | –5.45 ± 4.32 | 22.5 |  |
| [Pearce](https://pubmed.ncbi.nlm.nih.gov/?term=Pearce+IA&cauthor_id=9924328) IA (1998)^76^ | | USA | | RCT | Laser vs cryogherapy | 102 | 3 | -7.3, -2.2 | NA |
| Hwang CK (2015)^77^ | | USA | | Retrospective | Laser or Bevacizumab | 54 | 5 | −3.7 vs −10.1, 0.6 vs −4.7 | NA |
| Roohipoor R (2018)^78^ | | Iran | | Retrospective | Laser or Bevacizumab | 986 | 2 | -2.84±2.77 vs -1.26±3.19 | NA |
| [Pandiri](https://pubmed.ncbi.nlm.nih.gov/?term=Pandiri+S&cauthor_id=40662937) S (2025)^79^ | | UK | | Retrospective | Bevacizumab, Bevacizumab+ laser, laser | 100 | 4.5 | -4.28,-3.05, -7.46 | NA |
| Seo EJ (2018)^80^ | | Korea | | Retrospective | Bevacizumab+ laser vs laser | 75 | 10 | −0.43 ± 0.58 vs −0.94 ± 2.69 | 0 vs 2 |
| Yoon JM (2019)^81^ | | Korea | Retrospective | Laser vs laser + Bevacizumab | 101 | 1.5 | -4.62 ± 4.00, -5.53 ± 2.21, -1.40 ± 2.19 | 6, 6, 0 |  |
| Kiran Yenice E (2023)^82^ | | Turkey | Retrospective | Laser vs Bevacizumab | 86 | 1 | - 0.5 ± 2.0 vs 0.8 ± 1.7 | 1,1 |  |
| Araz-Ersan B (2015)^83^ | | Turkey | Prospective | Bevacizumab + laser | 18 | 2 | −0.15 ± 3.01 | NA |  |
| Celik G (2020)^84^ | | Turkey | Retrospective | Bevacizumab vs laser | 79 | 5 | 0.04 ± 1.52 vs -0.51 ± 3.75 | NA |  |
| Lolas M (2017)^85^ | | Chile | Prospective | Laser or Bevacizumab | 144 | 1 | -1.75 | 22 |  |
| Gunay M (2016)^86^ | | Turkey | Retrospective | Bevacizumab vs laser | 42 | 1 | 0.15, 0.75 | 7.4, 12.2 |  |
| Hoppe C (2022)^87^ | | USA | | Retrospective | Laser or Ranibizumab | 68 | 3 | -1.74 | 7 |
| Tiryaki Demir S (2021)^88^ | | Turkey | | Retrospective | Laser or Bevacizumab or laser + Bevacizumab | 160 | 3 | 0.46±1.93, 2.10±2.24, 0.88±2.31 | 0, 1, 0 |
| Chen YC (2020)^89^ | | Taiwan | Retrospective | Laser vs anti-VEGF | 47 | 9 | −3.49 ± 4.39 vs −0.16 ± 2.00 | NA |  |
| Adams GGW (2018)^90^ | | England | | Prospective | Laser or anti-VEGF | 327 | 1 | 0.44 (−1.3 to 1.3) | 50.7 |
| Lu X (2022)^91^ | | Japan | | RCT | Anti-VEGF vs Laser | 28 | 5 | -2.43±3.56 vs -0.53±3.12 | NA |
| Linghu DD (2022)^92^ | | China | | Retrospective | Anti-VEGF vs laser | 1627 | 2 | 1.8, 0.19 | 27.5 |
| Kang HG (2019)^93^ | | Korea | Retrospective | Laser vs anti-VEGF | 52 | 5 | -1.0 and +0.22 ± 3.00 | NA |  |
| Park SH (2023)^94^ | | Korea | Retrospective | Laser or anti-VEGF | 114 | 1.8 | − 1.33 ± 3.19 | NA |  |
| Chen YC (2018)^95^ | | Taiwan | Retrospective | Ranibizumab vs Bevacizumab | 62 | 3 | −0.65 ±3.83 vs −1.18 ± 0.89 | 0 vs16.7 |  |
| Kang HG (2018)^96^ | | Korea | Retrospective | Bevacizumab or ranibizumab | 153 | 3 | +0.10 ± 3.66 and +0.22 ± 3.00 | NA |  |
| Tung HF (2024)^97^ | | Taiwan | | Retrospective | Ranibizumab vs Bevacizumab | 60 | 3 | 0.32 ± 2.01 vs 0.32 ± 2.53 | 0.6 vs 0.8 |
| Suren E (2022)^98^ | | Turkey | | Retrospective | Bevacizumab, ranibizumab or aflibercept | 187 | 3 | − 2.53 ± 2.58 vs 0.28 ± 1.66 vs 0.62 ± 1.35 | NA |
| Gangwe AB (2021)^99^ | | India | RCT | Ranibizumab vs laser after ranibizumab | 63 | 0.5 | -1.0D ± 1.3 vs 0.5D ± 1.9 | NA |  |
| Gunay M (2017)^100^ | | Turkey | Retrospective | Bevacizumab, ranibizumab, laser | 264 | 1.5 | −0.57, 0.78, −0.81 | 12.7, 13.6, 14 |  |
| Murakami T (2023)^101^ | | Japan | Retrospective | Laser vs Bevacizumab or ranibizumab | 264 | 4 | −1.77 ± 3.31 vs −1.09 ± 3.68 | NA |  |
| [Agarkar](https://pubmed.ncbi.nlm.nih.gov/?term=Agarkar+S&cauthor_id=28905828) SD (2017)^102^ | | India | | Retrospective | Laser vs vitrectomy | 28 | 2 | -7.47 ± 1.38 vs -6.41±1.91 | NA |
| [Tachikawa](https://pubmed.ncbi.nlm.nih.gov/?term=Tachikawa+T&cauthor_id=32648074) T (2020)^103^ | | Japan | | Prospective | Laser vs vitrectomy | 161 | 3 | -3.31, -12 | 14.3 |

NA= not applicable
